# Improving primary health care quality for refugees and asylum seekers: A systematic review of interventional approaches

**DOI:** 10.1111/hex.13365

**Published:** 2021-10-15

**Authors:** Maha P. Iqbal, Ramesh Walpola, Ben Harris‐Roxas, Jiadai Li, Stephen Mears, John Hall, Reema Harrison

**Affiliations:** ^1^ School of Population Health, UNSW Medicine University of New South Wales Sydney New South Wales Australia; ^2^ School of Population Health University of New South Wales Sydney New South Wales Australia; ^3^ South Eastern Sydney Research Collaboration Hub (SEaRCH), Population and Community Health South Eastern Sydney Local Health District Darlinghurst New South Wales Australia; ^4^ Hunter New England Medical Library New Lambton New South Wales Australia; ^5^ Centre for Health Systems and Safety Research, Australian Institute of Health Innovation; Level 6, Faculty of Medicine, Health and Human Sciences Macquarie University Sydney New South Wales Australia

**Keywords:** asylum seekers, interventions, OECD (Organization for Economic Co‐Operation and Development) countries, primary health care, refugees, systematic review

## Abstract

**Background:**

It has been widely acknowledged that refugees are at risk of poorer health outcomes, spanning mental health and general well‐being. A common point of access to health care for the migrant population is via the primary health care network in the country of resettlement. This review aims to synthesize the evidence of primary health care interventions to improve the quality of health care provided to refugees and asylum seekers.

**Methods:**

A systematic review was undertaken, and 55 articles were included in the final review. The Preferred Reporting Items for Systematic Reviews was used to guide the reporting of the review, and articles were managed using a reference‐management software (Covidence). The findings were analysed using a narrative empirical synthesis. A quality assessment was conducted for all the studies included.

**Results:**

The interventions within the broad primary care setting could be organized into four categories, that is, those that focused on developing the skills of individual refugees/asylum seekers and their families; skills of primary health care workers; system and/or service integration models and structures; and lastly, interventions enhancing communication services. Promoting effective health care delivery for refugees, asylum seekers and their families is a complex challenge faced by primary care professionals, the patients themselves and the communication between them.

**Conclusion:**

This review highlights the innovative interventions in primary care promoting refugee health. Primary care interventions mostly focused on upskilling doctors, with a paucity of research exploring the involvement of other health care members. Further research can explore the involvement of interprofessional team members in providing effective refugee/migrant health.

**Patient or Public Contribution:**

Patient and public involvement was explored in terms of interventions designed to improve health care delivery for the humanitarian migrant population, that is, specifically refugees and asylum seekers.

## 1 BACKGROUND

Globally, the number of humanitarian migrants, who include refugees and displaced people, has been consistently increasing, with an unprecedented 70.8 million people around the world being forced to leave their home country in 2019 due to conflict and persecution.^1^ There are currently more displaced people who have left their current home or residence than at any point since reliable data have been recorded.^2^ The United Nations High Commissioner for Refugees (UNHCR) defines a refugee as a person ‘who is unable or unwilling to return to their country of origin owing to a well‐founded fear of being persecuted for reasons of race, religion, nationality, membership of a particular social group, or political opinion’ and an asylum‐seeker is ‘someone whose request for sanctuary has yet to be processed’.^3^ Therefore, an asylum seeker is seeking international protection, but whose claim for refugee status is yet to be determined. The UNHCR was originally established in 1950 to help the refugees of the Second World War,^4^ and developed the 1951 Refugee Convention to safeguard the rights of refugees. Although the health and well‐being of refugees were not specifically mentioned in the 1951 convention, the World Health Organization (WHO) Constitution ‘envisages… the highest attainable standard of health as a fundamental right of every human being’.^5^ As such, nation states that accept humanitarian migrants have a responsibility to ensure that the health and well‐being of this group are maintained throughout their resettlement process.

It has been widely acknowledged that specifically refugees and asylum seekers may experience poorer health outcomes, spanning mental health and general well‐being. This is due to a combination of factors including high burden of disease, poor health care, poverty and the hazards associated with migration.^6^ The literature also highlights that many displaced people are reluctant to seek health care assistance when needed due to multiple reasons including, but not limited to, cultural beliefs and psychological trauma. The humanitarian migrants are at risk of poor health outcomes, which is further compounded by reluctance to seek health care assistance when needed due to a range of complex factors.^7^, ^8^ Timely access to high‐quality care during resettlement is commonly reported as a challenge amongst refugee populations.^9^

The most commonly reported point of access to health care for migrants including refugees and asylum seekers is via the primary health care/community network in the country of resettlement.^10^ A recent systematic review has identified a number of constraints that limit the provision of quality health care to refugee populations including access to health care services, provision of focused care and further resettlement.^11^ Access to health care delivery is frequently identified as a barrier for effective health care for refugees and asylum seekers. Often, this is linked to the fragmented and difficult‐to‐navigate health care systems in countries of resettlement^12^ or the reluctance of refugees/asylum seekers to access health care for simple reasons like communication barriers.^13^ The review also outlined a number of aspects of care quality that should be targets for improvement to enhance health care and outcomes amongst refugees and displaced people. Some of these aspects include building a trusting relationship between patients and practitioners; improving communication; ensuring cultural and social awareness by the practitioners; and ensuring that there is sufficient time to address the needs of refugees.^11^ Promoting continuity of health care and ensuring adequate resources to promote this are also a key part of resettlement processes.^11^, ^13^ The resettlement process is one component contributing to complex care needs amongst refugees and asylum seekers. Complex care needs describe a diverse population who experiences a combination of medical conditions and requirements for long‐term care along with behavioural and/or social need.^14^ In the context of refugees and asylum seekers, complex care needs may comprise resettlement, social acclimatization and health concerns.^15^

Primary health care systems globally have explored and adopted numerous approaches to improve the quality of health care provided to refugees and asylum seekers, and yet, knowledge of the nature of the interventions used and their impacts is fragmented.^11^ The primary health care system is the entry level into the health system via which the people can enter the health system, and it includes a broad range of activities and services from health promotion and prevention to the treatment and management of acute and chronic conditions.^16^, ^17^ The present review therefore aims to synthesize the evidence on primary health care interventions to improve the quality of health care provided to refugees and asylum seekers. This review focuses on the interventions exclusively developed in primary care delivery for refugees and asylum seekers in OECD (Organization for Economic Co‐Operation and Development) countries of resettlement and to establish evidence of their impacts on care quality. These findings are valuable for health care providers and policy makers towards the systematic enhancement of the quality of health care provision to sustain the complex care needs of refugee and asylum seeker populations.

## 2 METHODS

A systematic review^18^, ^19^, ^20^ was undertaken and the Preferred Reporting Items for Systematic Reviews and Meta‐analysis (PRISMA) was used to guide the reporting of the review.^21^

### 2.1 Data sources and study strategy

The search strategy was developed in liaison with a medical information specialist (S. M.). A medical information specialist is a librarian (information specialist) who specializes in health and medical literature. This strategy was applied to the following five databases from inception till 2 September 2020 for relevant studies: CINAHL, EMBASE, MEDLINE, PsycINFO and Web of Science. Search terms were combined for primary health care, refugees and asylum seekers. All searches were limited to studies published in the English language only, but no date limits were applied. The detailed search strategy for the databases is attached as File S1.

#### 2.1.1 Eligibility criteria

##### Inclusion criteria

The eligibility criteria were developed using the Population, Intervention, Comparison and Outcome (PICO) framework.^19^ Articles that fulfilled the following criteria were included: (1) articles published in the English language; (2) empirical and original studies; (3) research conducted in the primary health care setting in countries of resettlement (OECD countries); and (4) articles reporting an intervention to enhance any of the six outcomes that meet the definition of health care quality: health care *safety*, *effectiveness of care*, *timeliness of care*, *efficiency of care*, *equitable* and *person‐centred care*. Quality of care was defined as that aligned with the six pillars of quality identified in the WHO's definition of quality of care: ‘the extent to which health care services provided to individuals and patient populations improve desired health outcomes’. To achieve this, health care must be ‘safe, effective, timely, efficient, equitable and people‐centred’.^22^

##### Exclusion criteria

Articles that reported interventions that did not occur in a primary care setting or include a component that occurred within a primary care setting were excluded, along with those that were not focused on the target population of refugees and/or asylum seekers. Articles that were commentary, opinion pieces, editorials and non‐peer‐reviewed were also excluded.

### 2.2 Study selection and data extraction

Articles were managed using a reference‐management software (Covidence), and duplicates were removed. The process of title and abstract screening was undertaken independently by two reviewers (M. P. I.; J. L.) to identify potentially relevant studies. The retained studies were subjected to a full‐text review in which the inclusion criteria were independently applied to the full‐text articles by two reviewers (M. P. I. and J. L.). Two other team members reviewed all the full‐text articles identified as fulfilling the inclusion criteria (R. H. and B. H.‐R.). The team then met to discuss any discrepancies with regard to eligibility in relation to the inclusion criteria and agreed on the final studies for inclusion.

The data extraction proforma was developed by the research team to address the review questions. The following study characteristics were extracted using the finalized proforma: investigators, year, country, setting, sample and background, design and health care professional involved in the delivery of the intervention and the intervention.

### 2.3 Assessment of quality

All the included articles were assessed and evaluated using the comprehensive Quality Appraisal for Diverse Studies (QuADS) tool, which is specifically designed to appraise qualitative, mixed and multimethod studies in health services research^23^ (see File S2). The nature of health services research involves diverse study designs that can be in‐depth qualitative studies, mixed methods and multimethod approaches of exploration and evaluation.^23^ Each criterion was scored on a 4‐point scale ranging from 0 to 3. The QuADS tool was independently applied to the studies by two reviewers (M. P. I.; J. L.). Discrepancies were discussed and resolved by a third reviewer (R. H.).

### 2.4 Data synthesis

The findings were analysed using a narrative empirical synthesis based on the aims of the systematic review.^24^ Narrative synthesis in systematic reviews is particularly useful in understanding the effects of the interventions as well as the factors that impact the implementation of interventions.^24^ The narrative approach was used to synthesize the qualitative and quantitative findings, which allowed in‐depth exploration and collective understanding from multiple studies that developed a broader perception of the phenomenon under study. The initial descriptions of eligible studies and results are tabulated in Table 1.

**Table 1:** Primary care interventions

Investigators	Year	Country	Setting	Sample and background	Design	Health care professional involved in delivery of intervention	Intervention
Balachandra et al.	2009	USA	Family‐centred maternity care	One Vietnamese couple	Illustrative case study with refugee	A complex team including interpreters, the perinatal case manager, the resident doctor and a faculty member	This intervention implemented an interprofessional, family‐centred antenatal care delivery model for deaf refugees. This was provided in the format of patient‐centred medical home visits with improved access to effective care
Ballard et al.	2018	USA	Primary health care	11 Karen Refugees and their children from Burma	Mixed methods	Marriage and family therapists, language interpreters and local health care workers provided the intervention	Implementing a family‐focused intervention (i.e., an educational module) that is a parenting intervention to manage children's misbehaviours in the context of trauma and relocation stress
					Qualitative and quantitative methods		
					Ethnographic interviews with caregivers and children as well as structured assessments at baseline and follow‐up		
					Psychological and relational standardized measures were administered at baseline, after participation in the intervention and at the 3‐month follow‐up		
Benjumea‐Bedoya et al.	2019	Canada	Primary care clinics	274 refugees from 23 countries were tested	Mixed methods	Physicians, nurse practitioners and primary care nurses ran the programme	This intervention is a free of cost, integrated intervention to access screening and treatment of refugees at BridgeCare Clinic for Latent Tuberculosis Infection (LTBI) screening and treatment. Free QuantiFERON‐TB Gold Interferon Gamma Release Assay (IGRA) testing and treatment were provided to clients
					Qualitative and quantitative methods		
Berkson et al.	2014	USA	Primary health care	126 Cambodian refugees	Quantitative methods	A mental health practitioner and a Cambodian community health worker	A holistic, culturally focused intervention to promote patient access to care and increased ability to adhere to healthy lifestyle modifications. The intervention was in the format of health promotion groups (i.e., 5 sessions) for Cambodian survivors of torture over the period 2007–2011. An American mental health practitioner and a Cambodian community health worker cofacilitated the health promotion groups (HPGs). The cofacilitators integrate Khmer health concepts with evidence‐based biomedicine and encourage participants to adopt an informed and integrated approach to their health
					Pre‐ and posttest Health Promotion Questionnaire (HPQ) translated into Khmer and administered in a semistructured interview by the Cambodian community health worker		
Biegler et al.	2016	USA & Canada	Primary health care	18 health providers participated (10 in the intervention group and 8 in the control group)	Randomized control trial	Primary care doctors	An innovative, online Health Information Technology (HIT) intervention was developed that has four components: (1) web‐based provider training, (2) multimedia electronic screening of depression and PTSD in the patients' primary language, (3) computer‐generated risk assessment scores delivered directly to the provider and (4) clinical decision support
					Quantitative methods		
				390 Cambodian refugees participated (272 patients in the intervention group and 118 patients in the control group)	Pre‐Hopkins Symptom checklist (HSC) and Harvard Trauma Questionnaire (HTQ) survey and a 12‐week follow‐up survey		
Birman et al.	2008	USA	Community‐based mental health service Primary health care	97 children and adolescents who were refugees participated	Quantitative	Doctors provided services and some mental health workers were also involved	This intervention is a collaborative, family‐centred, effective intervention designed to promote mental health among refugees. A multitude of services were offered including individual treatment, group treatment, family treatment, psychiatric services, case management, consultation, treatment and support services
					Used the Child and Adolescent Functional Assessment Scale (CAFAS) to rate the participants' functioning		
					The Trauma Event Checklist of Harvard Trauma Questionnaire (HTQ) was used to record the types and number of traumatic events		
Bonvicini et al.	2019	Italy	Primary health care	368 irregular immigrants, (i.e., immigrants who did not have a valid residence permit and who were therefore not entitled to choose a general practitioner.)	Retrospective cohort study‐ epidemiological method to collect data	Primary care doctors	This was a person‐centred, efficient intervention designed to improve the compliance with Tuberculosis Screening in Irregular Immigrants
Borgschulte et al.	2018	Germany	Primary care outpatient clinic	984 patient contacts were registered, mainly by young persons from Western Balkan countries and Syria	Mixed methods: Quantitative and qualitative methods	Doctors, nurses and social workers	This intervention was designed within the refugee accommodation and was in the format of an outpatient clinic to provide timely, person‐centred and equitable access to health care
					Questionnaires, interviews, and participatory observation		
Bosson et al.	2017	USA	Patient‐centred medical home approach to	525 out of 540 refugees completed the RHS‐15, and in 65 refugees (12.4%), there was a need for mental health services. 40 of these refugees entered the programme	Mixed methods	Clinical psychologists and psychiatrists provided individual mental health care and counselling. Clinical psychology graduate students were also included to assess and treat refugees	Interprofessional training model of care focused on mental trauma and to provide mental health assessment and consultation for refugees. The care was individualized for the refugee and included a variety of family and social/community contacts
			Primary health care		Refugee Health Screener 15 (RHS‐15)		
					Health care provider interview on their experience with the programme		
Bourne	2004	UK	African well Women's Clinic	1111 African women attended this service	A case study	Nurses provided the services	A patient‐centred intervention for patients with a specific health condition to improve improved access to care in terms of healthy lifestyle, screening and treatment
							Established African Well Women's Clinic for women who underwent Female Genital Mutilation (FGM) to provide culturally sensitive services. Health care services are provided to women to discuss their health concerns in specific languages, and the service also facilitates communication with the GP and direct referral to specialists
							This intervention is designed to be person centred
Browne et al.	2018	Canada	Primary care	Equipping Primary Health Care staff	Mixed methods	Primary care doctors, nurses and nurse practitioners, social workers, nutritionists, counsellors and, depending on the clinic, pharmacists, physiotherapists, dentists and Indigenous Elders	This intervention focused on promoting the delivery of safe, effective and equitable health care, in addition to effective health care delivery via an organizational‐level health equity intervention
				Equipping Primary Health Care staff No sample size specified	Quantitative and Qualitative		
					In‐depth, open‐ended interviews		
					Observations in each setting		
					Staff ratings of confidence in selected aspects of equity‐oriented health care at preintervention, posteducation and postintervention		
Bull et al.	2018	USA	Primary health care	68 patients were included in the project. 12 Bhutanese patients participated in group visits and 56 were included in standard care with 15 min appointments	Quantitative data were collected during the monthly group visits and growth parameters were reviewed by physicians. A one‐time postanonymous e‐survey was conducted after the intervention	The team involved family physicians, a paediatrician, a registered nurse (RN) and a Bhutanese Nepali interpreter	The intervention was designed to improve primary health care for refugee children, with a specific focus on failure to Thrive (FTT). The intervention was conducted via a series of culturally adapted group visits (GV) for patients with the same first language
Carter et al.	2017	USA	Primary care clinic for refugees	121 out of 436 refugees were latent tuberculous infection LTBI positive and 103 of them were referred to the pharmacist‐run LTBI clinic for treatment. The completion rate was 94%	Quantitative study	Pharmacists	A structured model delivered via a clinical pharmacist that focused efficiently on tracking patients and ensuring completion of screening and treatment of tuberculosis. The appointments were coordinated with the resettlement agency and transportation service was provided. This service was free of cost
					A retrospective chart review was conducted among refugees screened for LTBI and general information was collected		
				A clinical pharmacist‐run latent tuberculous infection LTBI clinic was established	Five‐week follow‐up visits were conducted		
Cheng et al.	2019	Australia	Integrated health care pathway	1087 were referred to the pathway, and 951 transitioned through the pathway. Refugees and asylum seekers were mainly from Afghanistan, Sri Lanka and Iran	Mixed methods: Quantitative and qualitative data	Allied health clinician, doctors and nurses involved in the triage	An interprofessional, efficient, effective health service was especially designed to provide easy access to refugees and asylum seekers. More specifically, a triage system was set up to link patients based on the care that they needed
					Data were collected during the screening process and the sessions		
					Brief exit surveys were conducted with clients and structured staff debriefing sessions were coordinated at the end of each triage session		
					Follow‐up phone calls were made for feedback and further needs		
Clabots and Dolphin	1992	USA	Community health care	A total of 378 tapes were made and sent to health services agencies; 8 agencies responded to the survey and sent results back	Quantitative	Online/videotapes	A person‐centred, health promotion intervention that is in the format of 9 multilingual educational videotapes in 7 languages to help immigrants and refugees improve their health literacy knowledge and understand how to gain access to the health care system
					An evaluation survey was required from the agencies who received tapes		A video tape was used in clinic waiting rooms, during the one to one teaching situations and for patients use at home
Culhane‐Pera et al.	2005	USA	Community health centres	39 participants who were Hmong refugees with type 1 diabetes mellitus with poor glycaemic control	Quantitative study	Family physician, diabetes nurse educator and nurse assistant, social worker and exercise specialists	A collaborative, holistic, person‐centred intervention promoting improved access to patients, with a focus on diabetes management of the patients.
					Analyses of pre‐ and postintervention measures of physical health, mental health and behaviour		Group visit structure involving check‐in, group discussions, one‐to‐one discussion and exercise
Duke and Brunger	2015	Canada	Family medicine	From 2006 to 2012, the patient numbers ranged between 21 and 107 refugee patients	Quantitative	Medical students conduct assessments of medical histories and basic physical screening, while working with an interpreter with supervision by a family doctor and settlement public health nurse. The refugee patients are then directed to a family physician	A collaborative, educational intervention promoting improved health care access to newly arrived refugees to gain health care access and at the same time provide opportunities and mentorship to medical students in the practice of multiculture health care
					Quantitative assessment of sessions completed, volunteer engagement, physician involvement, referrals and number of patients matched to physicians		
					A survey of physicians involved was conducted to collect physician perspectives on the strengths and challenges of the project		
Dutcher et al.	2008	USA	Health care delivery	No sample size reported	Quantitative—data on visiting the webpage	Online resource	An online, easy to access, person‐centred intervention providing a resource of health care information to refugees and asylum seekers‐ the Refugee Health Information Network (RHIN)
							RHIN currently focuses on providing quality materials to health providers who work with refugee clients.
Ekblad et al.	2013	Sweden	Primary health care	11 primary health clinicians used and evaluated the tool.	Mixed methods: Quantitative and qualitative data	Primary care doctors	This intervention was an online tool in the format of a virtual patient with a history of trauma to train primary health care professionals to provide safe and effective care to refugees with a history of trauma and refugee mental health
					Pre‐ and postquestionnaire		
					15–30 min telephone interviews		
Esala et al.	2018	USA	Integrated care	40 Karen Refugees were involved in the study	Qualitative exploration	Highly qualified psychotherapy and social work professionals who are supervised by senior providers with extensive experience working with traumatized refugees deliver the intervention	Timely, Integrated, effective behavioural health care intervention. This intervention is provided in the primary care health centre and provides psychotherapy and targeted case management services
					In‐depth, semistructured interviews were conducted		
Farokhi et al.	2014	USA	Primary health care	Majority of patients attending the clinic have been Nepali, Burmese, Iraqi, Iranian, Congolese, Burundi and Thai refugees	Qualitative data	Dental, medical and nursing students	An interprofessional, safe, educational intervention that involved students from dental, medical and nursing schools, under the mentorship of their faculty to serve the refugee community. The student‐run San Antonio Refugee Health Clinic (SARHC) was free of cost and provided an opportunity to train and educate students to serve the diverse refugee population
					Student perceptions about participating in this intervention were explored		
Ferrari et al.	2016	Canada	Primary health care	74 participated. 58 completed iCCAS in English, and 16 in Spanish	Mixed methods: Quantitative and qualitative	Family physicians and nurse practitioners	This intervention was an online, computer‐assisted client assessment tool completed by clients while waiting to see their family physician (FP) or nurse practitioner (NP)
					Exit experience survey among the clients, and Qualitative interviews with 9 family physicians (FPs) and nurse practitioners (NPs)		
Gondek et al.	2015	USA	Primary health care	A total of 14 sessions conducted with 348 participants. Varied ethnicity of patients: Middle Eastern (29.5%), Nepali (20.1%), Burmese and Thai (17.1%) and African (16.8%)	Quantitative study	An educational session had a breast cancer survivor as a speaker and a female physician to answer questions	A multilingual, person‐centred intervention that was designed to engage immigrant and Refugee Women in Breast Health Education. The intervention included breast cancer screening in a mobile mammography unit
					Pre‐ and posttest assessments were given during the training		
Goodkind	2005	USA	Integrated care	28 Hmong adults (majority women) and 27 undergraduate students participated in the intervention	A comprehensive, multimethod strategy, which included a within‐group longitudinal design with four data collection points and in‐depth qualitative recruitment and postintervention interviews	Undergraduate students	This intervention was a community‐based advocacy and learning programme for Hmong refugees. The intervention had two major components: (1) Learning Circles, which involved cultural exchange and one‐on‐one learning opportunities for Hmong adults, and (2) an advocacy component that involved undergraduates advocating for and transferring advocacy skills to Hmong families to increase their access to resources in their communities. This intervention was designed to promote person‐centred care and improved access to care by the refugees
Gould et al.	2010	Australia	Primary health care	76 patients received health assessment; 69 of them received the assessment within the first year of arrival	Mixed methods: Qualitative and quantitative methods and included a description of the service delivery model and a retrospective analysis of data from the records of the first 76 patients referred to the clinic for a comprehensive health assessment	The clinical team consists of a clinical nurse, consultant (CNC), administrative support, access to pathology and diagnostic imaging services and pharmaceuticals for the clinic. However, medical services at the clinic are provided by five general practitioners	This intervention was designed to provide effective, culturally appropriate and timely health services via an interprofessional team
Grigg‐Saito et al.	2010	USA	Community health centres	More than 1000 health professionals completed cultural competence and Cambodian health beliefs training. A sample of 297 professionals completed pretest and posttest evaluations	Quantitative data reported	Doctors, nurses, community health workers, religious personnel and translators	A comprehensive service model was implemented to address refugees' physical, psychosocial and spiritual needs. Many interprofessional groups were involved in each subprogramme like Buddhist monks' consultations, community health workers, peer leaders and teaching assistants and involvement of parents and community partners. This model was specifically designed to address health disparities in the Cambodian refugee and immigrant community of Lowell, MA
Jahn et al.	2018	Germany	Integrated care	No sample size suggested	A multisited qualitative study in 6 refugee centres in 5 cities	Doctors and nurses	This intervention was designed to improve communication between different health care sectors for refugees and asylum seekers. The intervention was a patient‐held personal health records (PHR) that was used in patient transfer between health sectors. A total of 11 physicians participated (10 physicians were located in the primary care and one was located in a psychosocial care centre). 6 nurses were involved in the intervention
					Interviews with physicians and survey nurses		
Jirovsky et al.	2018	European countries	Primary health care (PHC)	A total of 390 participants registered as health care professionals for the online course in 6 countries with	Quantitative	Online	An online, web‐based course containing 8 modules of information to support primary health care professionals in the provision of high‐quality care for refugees and migrants
					An online evaluation survey was conducted after the training. Pre‐ and postcompletion knowledge tests		The English template was translated into 7 languages in 6 countries
Johnson et al.	2006	UK	Local Somali community centre or in participants' home	Twenty Somalis were presented with three communication tools and were asked a set of general questions in Somali that they had to answer using each tool: (1) a paper‐based communication book containing symbols and bilingual text labels; (2) a laptop PC with a mouse pad containing the same symbols, text labels and augmented with digitized Somali speech; and (3) a tablet PC with touch screen containing the same software and digitized Somali speech	Qualitative study	Primary care doctors	This intervention utilized alternative communication strategies to communicate with both literate and illiterate Somalis, thus promoting safe and effective health care delivery
			Health care delivery		Video‐recorded interviews		
Kennedy et al.	1999	USA	Family medicine centre and Refugee Services Programme	More than 1600 refugees received assessments during the first 30 months of the programme	Case study Quantitative data	Family medicine faculty and residents perform all health assessment exams	A comprehensive refugee health screening programme was implemented to provide a single point of access for all family members of refugees/asylum seekers. A range of appropriate, interpreting services, comprehensive health assessments that include a thorough mental health screening, data collection and evaluation and education of health care providers to deliver culturally responsive care was made available
Kirmayer et al.	2003	Canada	Health care delivery	Collected data of the first 100 cases referred to cultural consultation service (CCS)	Mixed methods	Core CCS personnel included 2 part‐time psychiatrists, as well as psychologists, social workers, psychiatric nurses, medical anthropologists and trainees from these disciplines and from family medicine. A full‐time clinical psychologist acted as a clinical coordinator and triaged all referred cases	This intervention was designed to improve practitioner–patient communication in relation to a model of mental health service for multicultural societies. Three formats were available: the first one is a consultant + cultural expertise + patient; the second one is a consultant + cultural expertise; and the third one is a consultant + community organization
				102 referrals, People from 42 countries, speaking 28 languages, with more than 50 ethnocultural groups and 6 major religious traditions participated			
				29 clinicians completed service evaluation questionnaires; 86% were satisfied with the format	Mixed methods: Quantitative evaluation of the service involved assessing the outcome of consultations in terms of the following: 1) types of cases referred and evaluated, 2) use of specific professional and community resources, 3) types of interventions and recommendations, 4) the consulting clinician's satisfaction with the service and 5) the consulting clinician's concordance with the recommended interventions. The qualitative component of the evaluation included a participatory research model involving participant observation and observational data collection		
Martin et al.	2018	Australia	Primary health care	More than 300 sessions were delivered to 3000 participants from 2012 to 2016. Over 400 health care volunteers were involved	Case study	Volunteer health care professionals include doctors, nurses, midwives, dentists, physiotherapists, dieticians and medical students in their final year of study	This is a health promotion intervention focused on providing effective and person‐focused health care information to refugees and asylum seekers. Interactive, health education sessions are provided by volunteer health care professionals at the request of established community groups. Interactive education sessions, 60–90 min each
							2 volunteer health care professionals for every 5–15 participants in each session
McHenry et al.	2016	USA	Family medicine	Approximately 173 residents completed the pre‐ and postsurveys	Quantitative: Pre‐ and postintervention, self‐administered surveys were used to measure clinician's knowledge, attitudes and comfort. Some open‐ended questions were also included	Primary care doctors	This intervention was a brief educational module focused on cross‐cultural considerations when caring for Burmese refugees i.e., cultural considerations and specific health care needs
Michael et al.	2019	USA	Primary health care/integrated care	285 refugees were included in the study	Quantitative date: Single variable logistic nonlinear mixed models were used	Primary care doctors	This intervention was a collaborative‐developed novel algorithm that guided the process by which refugees establish care in patient‐centred medical homes (PCMHs)
				A total of 20 unique countries were represented among the 285 participants in the study, including 41% from Syria, 17% from the Democratic Republic of Congo, 7% from Chad and 5% from Iraq			
Muller et al.	2020	Germany	General practice	A pilot study (text run) was conducted with 36 patients of Syrian origin	Quantitative data: A digital and audio‐supported questionnaire was completed when the DCAT was finished	Online/web‐based tool	This communication tool was in the form of a Digital Communication Assistance Tool (DCAT) to obtain medical history from refugees and asylum seekers. The tool was designed with 19 different languages and dialects
Njeru et al.	2015	USA	Health care delivery	8 digital stories were created on topics like medication management, glucose self‐monitoring, physical activity and nutrition for diabetes. Each of the 8 storytellers was from the Somali and Latino communities with diabetes (4 from each group)	Community‐based participatory research (CBPR).	Community health workers and translators	An effective and a person‐focused intervention that used digital storytelling to provide diabetes‐related information to refugees and immigrants. This intervention was designed to aid participants in managing diabetes
					Qualitative study		
					6 focus groups with 4–9 people in each group (37 total) involved in 60–90 min workshops that were held for 6 weeks. Results from the workshops were collected and summarized into 8 stories		
Northwood et al.	2020	USA	Primary care	193 out of 214 participants completed a baseline and follow‐up assessment.	Pragmatic randomized control trial with a baseline and follow‐up assessment	Psychotherapist, clinical social worker and primary care doctor	This intervention was specifically designed for Karen refugees and related to providing intensive psychotherapy and case management for patients with major depression presenting to primary care
				Karen refugees Involved in the project			
Ong et al.	2010	UK	General practice	A total of 280 refugee doctors participated; 42 were involved in more than one programme	Quantitative survey	The refugee doctors involved were from 16 different countries: Iraq, Iran, Afghanistan and Pakistan are the most frequently represented	This intervention focused on promoting learning and training of refugee doctors by offering them clinical attachments, supernumerary 6‐month posts and general practitioner (GP) training in the host country. They were involved in health care delivery in their host countries
Parmentier et al.	2004	UK	Health care delivery	Give refugees with miner illness a voucher that can be used in the pharmacy to exchange OTC medicines. A total of 200 vouchers were given to 184 refugees in the 5‐month project. 264 items were collected by refugees	Quantitative study	Pharmacists	This intervention helped allied health care staff, that is, pharmacists to manage minor illnesses of refugees by offering over the counter (OTC) medicines, thus promoting effective and timely provision of health care
Percac‐Lima et al.	2013	USA	Community health centre	There were 188 refugees (36 Somali, 48 Arabic, 104 Serbo‐Croatian speaking), 2072 English‐speaking and 2014 Spanish‐speaking women eligible for breast cancer screening	Quantitative study	Patient navigators	This intervention was a tailored Patient Navigator (PN) programme that provided knowledge about breast cancer screening for refugee women and encouraged them to complete the screening
Pottie and Hostland	2007	Canada	Family medicine/refugee centre	This intervention was pilot‐tested on 5 refugee families (15 individuals) at a shelter	Qualitative study: Follow‐up semistructured face‐to‐face interviews with students, GPs and refugees	Medical students and primary care doctors	This was an educational intervention designed to enhance refugee health care delivery and cultural competence of effective health care for refugees and asylum seekers. The intervention had a wide variety of formats including internet‐based training modules, a self‐assessment quiz and workshops to increase competence in cultural matters. Both refugees and medical practitioners including students participated in the intervention. After attending the educational component, students had the experience of working with at least 1 refugee family at a shelter under the mentorship of a family physician. Students who completed this programme were also eligible for further electives at a refugee health clinic
Prescott et al.	2018	USA	Community‐based educational workshops	12 workshops were conducted for 282 refugees from 33 countries	Mixed methods: Quantitative and qualitative study	The workshops were held approximately once per month with Doctor of Pharmacy Students, alumni and a faculty member	This interactive intervention was person‐focused and promoted safe health care delivery by providing a medication health literacy programme for refugees and asylum seekers. Refugees were provided basic information about medications with the help of laminated slides and demonstration kits
					Pre‐ and postquestions/surveys were conducted related to the content.		
					Brief, semistructured interviews of the refugees		
Reavy et al.	2012	USA	Community health centre	13 Clinic Health Advisors and 227 prenatal and paediatric refugee patients received assistance from a health advisor who spoke the refugees' preferred spoken language and English	Mixed methods: Qualitative and quantitative	Family practice physicians, certified nurse midwives, a paediatric nurse practitioner, registered nurses, a licensed case social worker, a dietician, medical assistants and office staff	This intervention was a new clinic model for prenatal and paediatric refugee patients, which is the C.A.R.E. (Culturally Appropriate Resources and Education) model that aimed to promote effective and efficient health care delivery
					Qualitative data were collected by observations, focus groups and individual interviews with advisors and the health care team		
					Quantitative data were collected from retrospective chart reviews to assess the outcome of patient health care		
Rodriguez‐Torres et al.	2019	USA	Primary health care	*N* = 126 Refugee women received the intervention	Quantitative methods	Patient navigator	A culturally Tailored, patient‐focused Patient Navigation (PN) Programme to increase Breast Cancer Screening in Refugee Women
							This intervention is designed to be person‐centred, providing improved access to care and with effective care provision
Schulz et al.	2014	Australia	General practice	20 consultations	Quantitative study	General practitioner and nurse	This intervention was a new telehealth clinic that promoted timely access to specialist care at the general practice along with the general practitioner and/or the practice nurse
Spruijt et al.	2020	The Netherlands	Primary health care	A total of 904 Eritrean migrants participated, and 401 of them attended the Latent Tuberculosis infection (LBTI) education programme and 257 received LTBI screening	Mixed methods	Primary care physician, nurse and technical assistant	This was a person‐centred, effective intervention designed to motivate migrant communities that are at high risk for TB to participate in a latent tuberculosis infection screening programme
					Questionnaires, consultations, semistructured group interviews and individual interviews		TB and LTBI screening and treatment activities were offered in multiple formats and consisted of three components: (1) TB and LTBI education, (2) LTBI screening and (3) LTBI treatment. The education and written materials were provided in Tigrinya, the Eritrean mother tongue
Sundquist et al.	2010	Sweden	Primary health care	243 refugee women participated from two locations, 131 in the intervention group and 112 in the control group	Quantitative study	The female leaders/instructors were either physiotherapists or physical education teachers	An effective intervention was developed in the format of a Primary health care‐based cardiorespiratory fitness programme for refugee women
				First‐generation refugees in Sweden from either the Middle East or Latin America.			
				(Refugee women recruited from two locations; one group was set as the intervention group and the other one was set as the control group).			The trial of the intervention compared the cardiorespiratory effect of a 6‐month primary health care‐ and community‐based exercise programme with a written prescription of exercise guide on refugee women with low activity
Teunissen et al.	2017	Five European countries: Austria, England, Greece, Ireland and the Netherlands	Primary health care	66 stakeholders participated in 62 Participatory Learning and Action (PLA) style groups. To develop supportive evidence‐based guidelines and training initiatives (G/TIs)	Qualitative study and observational data collection	General practitioners (GPs), practice nurses, receptionists, practice assistants, practice managers, academics and interpreters	This was a comprehensive, widely integrated intervention that promoted the implementation of person care guidelines and training initiatives to improve cross‐cultural communication in primary care consultations, especially with refugees and asylum seekers
				Stakeholders including migrant representatives, general practitioners (GPs), practice nurses, receptionists, practice assistants, practice managers, academics, interpreters, health service planners and policy makers			
Timlin et al.	2020	Australia	Primary health care	57 staff from 25 GP clinics participated. 95%, n = 54) were GPs and the remaining 5% were practice nurses	Quantitative data Rigorous record keeping, pre‐ and post‐practice assessments guided by a self‐reported 12‐point checklist, participant feedback	General practitioners and nurses	This collaborative, educational intervention, delivered by the practice facilitator, focused on developing the skills of general practice health care workers in the provision of safe and effective health care for refugees and asylum seekers
Wagner et al.	2015	USA	Primary health care	114 out of 140 participants, who were Cambodian refugees, completed the 1‐year assessments	Quantitative study	Community health care workers	A person‐centred, effective, community health worker (CHW)‐ delivered lifestyle intervention for the prevention of cardiometabolic disease, called Eat, Walk, Sleep (EWS) for Cambodian American refugees
Weissman et al.	2012	USA	Primary health care/health care delivery	None specified	Case study	Students and primary care doctors	This intervention discussed the free of cost, student‐run health initiatives provided by Refugee Health Partner (RHP) programme. The programmes offered vaccine clinics and health fairs; English as a second language classes in every Saturday afternoon; refugee education partners; mental health assistance; and promotion of women's health
Wieland et al.	2017	USA	Primary health care	25 refugees (15 Latino, 10 Somali) with type 2 diabetes mellitus were involved	Mixed methods: Qualitative data and Quantitative assessment and comparison of HBA1C	Online/digital tool	A person‐centred, effective intervention was developed that was in the format of a digital storytelling module targeting immigrants and refugees with type 2 diabetes mellitus (T2DM)
					Face‐to‐face structured interview and survey after the intervention		
					Follow‐up checking A1C level		A 12‐min culturally and linguistically tailored video included an introduction, four stories and an educational summary
Wittick et al.	2018	Australia	Primary health care	An Australian Refugee Health Practice Guide website	Qualitative study—semistructured interviews	General practitioners	This intervention was in the format of an online resource for general practitioners (GPs) to promote their role in supporting refugee health care in Australian general practice
				10 GP participated			
Yacoob et al.	2020	USA	Primary health care	Refugee patients (*n* = 171) were study sample	Quantitative study	Nurses and medical assistants	A lecture‐based educational intervention for nurses and medical assistants to enhance the vaccination uptake among high‐risk populations
Zehetmair et al.	2018	German	Reception centre	During the study period, a total of 86 imaginative stabilization group therapy sessions took place and *N* = 46 participants (Sub‐Saharan Africa, Middle East, South Asia and North‐Africa) visited the sessions at least once	Mixed methods: Quantitative study and qualitative exploration	Psychotherapists and a doctoral student of behavioural therapy	This was a person‐focused intervention design in the format of a psychotherapeutic group for traumatized male refugees. The programme used imaginative stabilization techniques to promote the mental health of participants
					Pre‐ and post self‐report questionnaires		
					Follow‐up interviews with 25 participants 2 weeks after the last session.		

## 3 RESULTS

### 3.1 Search results

**Figure 1:**
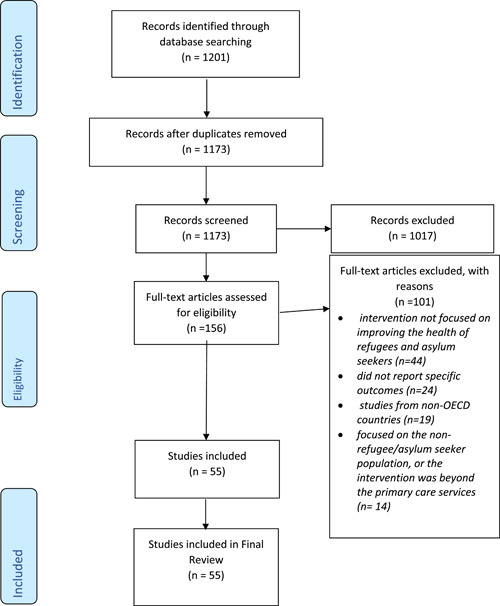
PRISMA flow diagram for a systematic review of the literature to explore interventions focusing on improving health care quality for refugees and asylum seekers in the context of primary health care. PRISMA, Preferred Reporting Items for Systematic Reviews

The systematic database search identified 1201 articles. After removal of duplicates, 1173 articles remained. A total of 1017 articles were excluded based on the title and abstract. The full text was reviewed of the remaining 156 articles, and these were assessed against the inclusion criteria. After this review, a total of 55 final articles were included in the narrative synthesis (Figure 1: PRISMA Flowchart).

### 3.2 Excluded studies

Studies (*n* = 101) were excluded at the full‐text review stage because they did not fulfil the inclusion criteria and for the following reasons: *n* = 44 reported an intervention that is not focused on improving the quality of care for refugees and asylum seekers, and *n* = 24 did not report eligible outcomes relevant to the inclusion criteria. In addition to these, *n* = 19 were studies from non‐OECD countries; *n* = 14 focused on the nonrefugee/asylum seeker population or the intervention was beyond the primary care context.

### 3.3 Study quality

The studies rated highly on a clear statement of research aims and appropriate study design descriptions to address the stated aims, and yet, generally received an average or low score for description of the data collection methods.^25^, ^26^, ^27^, ^28^, ^29^, ^30^, ^31^, ^32^, ^33^, ^34^, ^35^, ^36^, ^37^, ^38^, ^39^, ^40^, ^41^, ^42^, ^43^, ^44^ The majority of the studies received low scores (0–1/3) on criteria related to sampling^25^, ^27^, ^28^, ^29^, ^30^, ^31^, ^32^, ^33^, ^34^, ^35^, ^36^, ^37^, ^38^, ^39^, ^40^, ^41^, ^42^, ^43^, ^44^, ^45^, ^46^, ^47^, ^48^, ^49^, ^50^, ^51^, ^52^, ^53^, ^54^, ^55^, ^56^, ^57^, ^58^, ^59^, ^60^, ^61^, ^62^, ^63^, ^64^, ^65^, ^66^, ^67^, ^68^ and evidence of research stakeholders' involvement in the research design and conduct (0–1/3). We did not exclude studies based on the quality assessment; rather, the quality assessment data were used simply to indicate the strength of the available evidence.

### 3.4 Characteristics of the included studies

**Figure 2:**
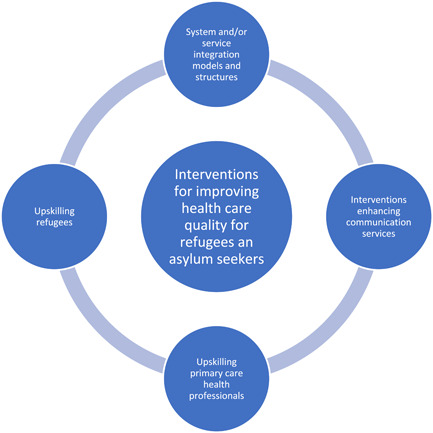
Interventions present within the primary care network that aim to improve the health care quality of refugees and asylum seekers in countries of resettlement

Of the total of 55 studies included in the review, the majority *n* = 35 were from North America (United States and Canada)^25^, ^26^, ^27^, ^28^, ^29^, ^30^, ^34^, ^35^, ^37^, ^39^, ^40^, ^41^, ^42^, ^44^, ^45^, ^46^, ^47^, ^49^, ^51^, ^52^, ^54^, ^55^, ^56^, ^57^, ^59^, ^62^, ^65^, ^66^, ^69^, ^70^, ^71^, ^72^, ^73^, ^74^, ^75^; 14 were from countries in Europe including the United Kingdom, the Netherlands, Germany, Sweden and Italy^31^, ^32^, ^33^, ^38^, ^48^, ^53^, ^60^, ^61^, ^63^, ^64^, ^68^, ^76^, ^77^, ^78^; and 6 were from Australia.^36^, ^43^, ^50^, ^58^, ^67^, ^79^ All articles were published between 1992 and 2020. The interventions, identified in this review, focused on four broad areas: (1) developing skills amongst individual refugees/asylum seekers and their families; (2) skill development of primary health care workers; (3) system and/or service integration models and structures; and (4) interventions enhancing communication services (Figure 2). Two interventions (2/55 studies) were organized to be included in more than one category outlined above.^29^, ^78^

The majority of the studies (29/55 studies) identified in this review discussed the involvement of doctors engaging with the interventions.^25^, ^26^, ^27^, ^28^, ^29^, ^31^, ^32^, ^34^, ^35^, ^36^, ^37^, ^41^, ^43^, ^46^, ^47^, ^50^, ^53^, ^56^, ^58^, ^67^, ^69^, ^70^, ^74^, ^76^, ^77^, ^78^, ^79^ Health care professionals involved in the interventions also included nurses or nurse practitioners (11/55 studies),^26^, ^28^, ^29^, ^31^, ^35^, ^36^, ^48^, ^50^, ^58^, ^77^, ^78^ undergraduate students (6/55 studies),^29^, ^30^, ^36^, ^40^, ^57^, ^78^ patient navigator roles/community health workers (4/55 studies),^39^, ^42^, ^44^, ^46^ clinical psychologists (4/55 studies),^35^, ^47^, ^54^, ^74^ pharmacists (3/55 studies),^38^, ^62^, ^71^ physiotherapists and/or exercise specialists (3/55 studies),^28^, ^36^, ^64^ a health care advisor or a refugee health facilitator role (2/55 studies),^41^, ^79^ midwives (1/55 studies),^36^ dentists (1/55 studies)^36^ and family therapists (1/55 studies).^45^ Some interventions were self‐directed and were online and/or computer based (10/55 studies).^33^, ^52^, ^53^, ^55^, ^57^, ^60^, ^66^, ^67^, ^69^, ^72^ A variety of study designs and methods were used including qualitative interviews (in‐depth, semistructured and unstructured; 24/55 studies)^26^, ^30^, ^31^, ^33^, ^40^, ^41^, ^45^, ^47^, ^53^, ^54^, ^55^, ^57^, ^62^, ^63^, ^66^, ^67^, ^68^, ^70^, ^73^, ^77^, ^78^; 20/55 studies reported quantitative information on administrative data sets from self‐reported surveys (collecting data on hospital/emergency visits and uptake of screening/treatment/vaccination)^26^, ^29^, ^30^, ^34^, ^36^, ^38^, ^39^, ^41^, ^43^, ^48^, ^49^, ^56^, ^58^, ^63^, ^66^, ^68^, ^69^, ^72^, ^75^, ^76^ and quantitative surveys (16/55 studies).^27^, ^29^, ^35^, ^42^, ^44^, ^47^, ^50^, ^51^, ^55^, ^57^, ^59^, ^60^, ^61^, ^62^, ^70^, ^77^ Some studies (9/55 studies) gathered baseline and postintervention data.^32^, ^37^, ^45^, ^46^, ^53^, ^56^, ^68^, ^71^, ^74^, ^79^ One study (1/55 study) reported researcher field reports collecting observational data.^78^ Few longitudinal studies (4/55 studies) were conducted, ranging over a period of 6 months,^57^, ^75^ 19 months^78^ and 3 years.^27^ A case method approach was adopted for four studies (4/55 studies).^25^, ^34^, ^36^, ^48^

(1) *Skills of individual refugees/asylum seekers and their families*: Twenty studies (20/55 studies) in the review focused on developing the skills of refugees, asylum seekers and their families. A substantial group of studies (*n* = 14) described health promotion interventions^28^, ^36^, ^39^, ^42^, ^44^, ^46^, ^51^, ^52^, ^56^, ^57^, ^63^, ^64^, ^66^, ^73^ for refugees/asylum seekers and their families and these were predominantly aimed at promoting access to the services available (five studies),^36^, ^46^, ^51^, ^52^, ^57^ improving engagement with and adherence to health regimes for better health care outcomes (seven studies).^28^, ^39^, ^42^, ^56^, ^63^, ^66^, ^73^ Two studies in this category were also designed to promote information about physical health and well‐being especially related to cardiovascular health like healthy diet, sleep and exercise.^44^, ^64^
  Six studies sought to advance the skills and ability of refugees and asylum seekers to talk about their health and health care with health professionals and broader health and social care workers.^29^, ^45^, ^47^, ^48^, ^54^, ^62^ The studies tackled a range of issues addressing mental health concerns (2/6 studies)^47^, ^54^ and trauma care related to past experiences and/or migration to a new country (3/6 studies).^45^, ^48^, ^54^ One intervention was more broadly seeking to improve individual patients' ability to speak with health care workers in the host country's health care system.^29^ Interventions often focused on a specific cultural and ethnic group.
  A variety of different formats of refugee training were discussed including face‐to‐face, small‐group, one‐to‐one patient counselling^54^, ^57^ and workshop formats^36^, ^45^, ^46^, ^63^; a single intervention often had multiple delivery formats. Educational toolkits with pictures, stories, video recordings and/or reading and web‐based resources were used in several interventions.^44^, ^52^, ^63^, ^73^ Small‐group, face‐to‐face sessions were generally identified as preferable as opposed to receiving resource packs because they promoted active engagement with participants that supported knowledge and skill development.^36^, ^63^ Several studies reported the involvement of trained language translators to ensure clarity in communication with patients and others offered bilingual/multilingual formats (*n* = 3 studies).^44^, ^45^, ^51^ The content of the interventions was also tailored to meet their cultural traditions and habits, by offering advice about dietary recommendations and especially when communicating about the general concept of well‐being (*n* = 2 studies).^44^, ^66^
  The interventions in this section reported improvement in several quality‐of‐care outcomes. Educational interventions (four studies) not only improved the ability of patients to communicate with health care professionals within the health system but also enhanced their access to it.^45^, ^46^, ^47^, ^48^ Participants' (two studies) access to health care was further promoted by offering timely, targeted and comprehensive health assessment models.^29^, ^54^, ^57^ For example, medical students were involved in providing comprehensive patient assessments and triaged them to appropriate care services under the mentorship of senior general practitioners (GPs).^29^ Participants' engagement with the interventions was further promoted via offering patient target interventions to a specific patient group and/or health conditions (eight studies)^28^, ^44^, ^48^, ^54^, ^57^, ^63^, ^64^, ^66^ by offering information in multiple languages (five studies)^42^, ^51^, ^52^, ^56^, ^62^ and in a multiple format (one study).^73^
(2) *Skill development of primary health care workers*: Fifteen studies (15/55 studies) outlined interventions to enhance the capacity and capability of primary health care providers to respond effectively to the complex care needs of the refugees. Interventions in this category were focused (eight studies) on promoting safe care delivery^30^, ^32^, ^53^, ^67^, ^70^, ^75^, ^78^, ^79^ and on effective health care delivery (12 studies),^29^, ^30^, ^32^, ^37^, ^49^, ^53^, ^67^, ^69^, ^70^, ^75^, ^78^, ^79^ while some (four studies) focused on provision of person‐centred care^40^, ^49^, ^61^, ^69^ and promoted equity‐oriented care (four studies).^32^, ^37^, ^53^, ^70^ Five interventions in this group primarily focused on a specific ethnic group and/or a health condition(s).^37^, ^49^, ^53^, ^61^, ^69^
  Eight interventions upskilling health care professional staff in care delivery for refugees and asylum seekers focused on safe health care practices,^32^, ^37^, ^53^, ^67^, ^69^, ^70^, ^78^, ^79^ out of which four studies also promoted equitable care delivery^32^, ^37^, ^53^, ^70^ with a range of primary health care professionals including GPs, nurses and other allied health staff. The nature and format of the interventions varied. Activities included health care professionals attending structured face‐to‐face workshops on topics like trauma‐ and violence‐informed care,^37^, ^70^, ^78^ personalized, face‐to‐face training in general practices,^79^ group discussions^70^ and online/web‐based education modules,^32^, ^67^, ^69^ asynchronous virtual patient encounters for doctors^53^ and clinician support tools to aid in screening and diagnostics.^69^ Three of the studies addressing equitable health care delivery reported interventions specifically designed to enhance the cultural immersion and competence of health care professionals for specific refugee/asylum seeker patient groups.^32^, ^37^, ^53^ Strategies varied from using virtual modes to simulate working with refugees who have experienced trauma^53^ to online modules to upskill GPs about cross‑cultural considerations for specific refugee populations.^32^, ^37^ Another study on promoting equitable health care delivery was in the format of face‐to‐face teaching, discussion groups and implementation of organizational structures to promote equity in care delivery.^70^ Posttraining surveys reported that health care professionals felt more confident in clinical encounters and were more likely to involve patients in future care discussions.^70^
  One study discussed the role of a refugee health fellow in building the capacity of primary health care professionals, including GPs and practice nurses, in providing effective health care for refugees and asylum seekers in an Australian context.^79^ The role of the facilitator was to identify and contact general practices involved in providing care to refugees and asylum seekers. Visits to the general practices aided in providing health resources, tools and frameworks to promote provision of safe and patient‐focused health care for refugees and asylum seekers.^79^ Moreover, multiple subsequent visits were arranged to discuss practice‐specific issues in relation to providing ongoing assistance. Tailored educational strategies were collaboratively developed by the fellow along with the GPs and practice nurses to deal with health care issues pertinent to refugees and asylum seekers. This targeted approach of developing skills of the general practice staff members was identified to bring about a positive change in the practitioner–patient clinical encounter with early identification of refugees and asylum seeker patient groups within the general practice. It was identified on self‐perception surveys, completed by participants before and after the implementation of the role, that there was an increased referral to mental health services and improvement in the provision of tailored care in terms of relevant screening and investigations.^79^ Another intervention was developed and adapted across several countries in Europe (Austria, England, Ireland, Greece and the Netherlands), tailored to their primary health care systems and targeted to their specific refugee populations.^78^ This intervention was in the form of supportive, evidence‐based guidelines and training initiatives to improve cross‐cultural communication between practice staff (i.e., including GPs, practice nurses, receptionists, practice assistants, practice managers, interpreters and the migrant patients.^78^ The positive outcomes, generally reported via in‐depth interviews such as self‐perceptions on improved communications with the refugees/asylum seekers coming into the practice, improved diagnoses and increased the confidence of general practitioners in developing management plans. The challenges that were reported varied according to the countries and their general practice structure. For example, in Ireland and Greece, the lack of structural resources to provide interpreters and logistic challenges with difficulties accessing trained interpreters created barriers in effective care provision.^78^
  Three interventions promoting person‐centred care targeted impact in distinct groups of refugees and asylum seekers. Two interventions were designed for a specific refugee/asylum seeker cultural group^49^, ^69^ and/or a specific health condition.^49^, ^69^ The effectiveness of these interventions was reported through patient self‐reported outcomes such as patient satisfaction in the overall quality of their mental health care, satisfaction with the primary care provider and the degree of patient‐centredness.^49^, ^69^ A statistical increase in the number of clinic visits for age‑appropriate child checks was reported, and increased health care professional satisfaction and confidence was measured in relation to providing care for a specific health condition like failure to thrive in refugee children.^49^ Another unique initiative promoting person‐centred care and improved access to health care involved upskilling refugees who were doctors themselves to become effective members of the National Health Service team, in the primary care delivery context, of the host country, that is, United Kingdom, and involve them in care delivery of the refugee population.^61^ Participating refugee doctors became familiar with the health care delivery standards of the host country and over 50% continued to work as doctors, providing care in the community.
  Seven interventions focused on specifically enhancing access to health care services amongst refugees by upskilling health care professionals.^29^, ^30^, ^32^, ^40^, ^67^, ^70^ Interventions (*n* = 2 studies) in this category were designed to upskill primary health care professionals in terms of the legal aspects for refugee health and approaches that orient refugees to a new health system to promote cultural safety and access to care in the host country.^32^, ^67^ For example, specific modules were designed with information on the different aspects of health care delivery and legal aspects such as the involvement of interpreters, translators and cultural mediators in care provision.^32^ A further study reported conducting face‐to‐face workshops on specific topics (such as trauma‐ and violence‐informed care) with general group discussions about issues raised by primary care professionals and online education modules to support harm reduction.^70^ Participants in these interventions reported gaining a better insight into the sensitive issues pertaining to migrant patients and gained improved knowledge about how to carefully navigate health care delivery for refugees and asylum seekers.^32^, ^67^, ^70^ Four studies reported interventions to develop the skills of health care students in the primary care delivery context by providing them opportunities to both learn from and experience cross‐cultural patient practice in training under the mentorship of senior health care professionals.^29^, ^30^, ^40^, ^65^ These interventions were designed to upskill prospective, primary health care staff in health care delivery of refugees. A framework was also developed to provide an initial access to health care for refugee patients and to refer them to appropriate health care services.^29^, ^65^ One study reported the design of an online module with workshops for students to attend before their engagement with refugee health clinics.^40^ This model was well received by students, primary care doctors and refugees because it facilitated easier access to health care and provided an opportunity to gain insight into the health care delivery of the host country.^40^ Similar to this model, Farokhi et al.^30^ presented a more interprofessional model involving nursing and dental students in addition to medical students. This approach of involving students in refugee care provision was reported to be an effective approach of developing the students' skills and knowledge of sensitive issues in providing care to refugees. The involvement and mentoring of students were rewarding experiences for the entire team including the senior health care professionals.^30^ Interprofessional models of care discussed have initiated holistic and accessible health care for the refugees.
(3) *System and/or service integration models and structures*: Seventeen interventions (17/55 studies) were designed to promote health system integration and continuity of care arrangements. Interventions in this subcategory were designed to improve different facets of health care delivery. However, all 17 studies focused on the delivery of safe health care.^25^, ^26^, ^27^, ^34^, ^35^, ^38^, ^41^, ^50^, ^58^, ^59^, ^68^, ^71^, ^72^, ^74^, ^76^, ^77^, ^78^ Some studies were designed to improve more than one health care outcome: Fourteen studies (14/17 studies) sought to enhance or enable delivery of person‐centred care,^26^, ^27^, ^33^, ^34^, ^35^, ^41^, ^43^, ^47^, ^58^, ^59^, ^60^, ^74^, ^76^, ^77^, ^78^ nine studies (9/17 studies) focused on promoting efficient care delivery^38^, ^41^, ^50^, ^58^, ^59^, ^68^, ^71^, ^76^, ^77^ and two studies (2/17 studies) enhanced equitable health care delivery.^25^, ^72^
  Six studies were designed to be in close proximity to the residences of refugees and asylum seekers, which promoted better engagement with them and aided in providing them timely access to care.^41^, ^50^, ^58^, ^59^, ^68^, ^77^ Collaborative models of care were also described that linked refugees and asylum seekers to appropriate health care facilities (*n* = 3 studies), such as patient‐centred medical homes for provision of health care by collaborative, interprofessional health care staff.^25^, ^72^ These collaborative models reported their effectiveness of health care provision in terms of measured health care outcomes^25^ such as enhanced timeliness of effective care provision reported in terms of reduced visits to emergency care.^72^
  Enhanced models of care were introduced and evaluated in seven studies from Europe,^77^, ^78^ the United States^41^, ^59^, ^72^ and Australia.^43^, ^50^ These interventions focused on enhanced patient access, provision of culturally oriented, family/person‐focused collaborative care in primary care more generally^43^, ^50^, ^59^, ^72^, ^77^, ^78^ and, more specifically, antenatal/maternity care.^25^, ^41^ These care models, implemented across different countries, aided in enhancing communication between health care professionals and patients, with enhanced delivery of timely and appropriate continuity of care. For example, the Integrated Healthcare Pathway triaged and linked patients within three weeks of arrival into the host country. Similar to this approach, Michael et al.^72^ discussed the development of an algorithm to link refugees to appropriate care models. The feasible location of the health care service near the refugee population and provision of care in multiple languages and formats improved both access to care and person/family‐focused health care delivery (three studies).^43^, ^77^, ^78^ In addition to these, Grigg‐Saito et al.^59^ presented a comprehensive ‘whole community model’ with collaborative community networks across multiple centres. These were involved in providing a range of holistic, support mechanisms, which include mental well‐being, physical health, youth development, communication and delivery of emotional empowerment interventions that are culturally appropriate for the Cambodian refugee patient community. A longitudinal assessment at baseline and postimplementation (measured after 1 year) of this comprehensive intervention outlined improvement in the mental health of participants, specifically resulting in reduced depression and anxiety. Moreover, there was an improvement in physical measures like blood sugar and blood pressure control, increased compliance with medications and improved awareness of health and well‐being.^59^
  This category included comprehensive care delivery models for mental health care (*n* = 6 studies) offered in accessible locations including homes, community centres and schools.^27^, ^35^, ^47^, ^55^, ^59^, ^68^, ^74^ Two studies discussed a family care approach promoting the mental and psychological well‐being of the entire family.^27^, ^59^ Four studies offered patients access to evidence‐based, trauma‐informed mental health care in the primary care clinical context itself, and these care services offered integration with wider sustainable social support networks.^35^, ^47^, ^68^, ^74^
  Patient access to care and person‐centred care within existing health services was promoted via interventions offering client information in multiple languages, translation services and also various formats (11 studies)^26^, ^27^, ^33^, ^34^, ^35^, ^43^, ^58^, ^59^, ^60^, ^74^, ^77^ or the involvement of cultural mediators in its delivery (five studies).^35^, ^41^, ^47^, ^76^, ^78^ Some of the interventions highlighted the free‐of‐cost services, which again related to improved accessibility to care and equitable health care for refugees and asylum seekers (*n* = 4 studies).^38^, ^71^, ^76^, ^77^ Distinct, patient‐focused and integrated clinics were also evaluated relative to specific diseases to enhance the provision of screening services, education and treatment for infectious diseases such as latent tuberculosis infections (two studies).^26^, ^76^ Some studies reported the involvement of allied health staff members (two studies) such as pharmacists in effectively leading these clinics for tuberculosis in particular^71^ and for other minor ailments.^38^ The studies reported on factors such as the importance of cultural mediators, language translators and cost‐free services in improving safety, quality, equity and accessibility to care and provision of person‐centred care.
(4) *Interventions enhancing communication systems*: Five studies (5/55 studies) assessed the use of interventions in health systems to enhance the communication aspect of health care delivery.^31^, ^33^, ^43^, ^55^, ^60^ The studies varied from interventions that enhanced communication between patients and practitioners (*n* = 3 studies)^33^, ^55^, ^60^ to those that promoted continuity and access to care via models that aided in communication between different health sectors even beyond primary care (*n* = 2 studies).^31^, ^43^ Interventions enhancing communication between the patient and the health care provider included online/digital, multimodal, multilingual tools that aided in capturing patient history and mental health screening.^33^, ^55^, ^60^ In addition to this, patient held, personal health records were implemented in primary care to improve communication between different sectors of health care delivery in the host country.^31^ Another model linked primary care to specialist care via the telehealth clinic option, in which refugees, along with their general practitioner or practice nurse in the primary care centre, could access and communicate with a specialist at the tertiary health care centre.^43^ This intervention was reported to facilitate enhanced communication and immediate transfer of information to the primary care doctor. Interventions outlined in this category were a system‐levels approach in improving communication aspects of care delivery for refugees and asylum seekers.

## 4 DISCUSSION

A systematic and structured approach was used to explore the primary health care interventions that were developed to optimize health care quality for refugees and asylum seekers in countries of resettlement. The WHO acknowledges the importance of promoting quality health care delivery for refugees and migrants because they are known to positively contribute to the country of resettlement, provided that they are in good health.^2^ Moreover, addressing the health needs of refugees and migrants early via preventive and primary care actually reduces long‐term costs for the health care system.^2^ Fifty‐five studies were identified and included. The included studies presented interventions closely associated with the WHO priority list for promoting refugee health.^80^ Interventions predominantly sought to enhance the skills of individual refugees/asylum seekers and their families to contribute to their own care and improve its quality. While some interventions sought to enhance the skills of primary health care workers to provide high‐quality care for humanitarian migrants, new models of care or approaches to promote system and/or service integration, communication and care arrangements were also identified. These interventions were not only limited to direct provision of health care delivery but also had wider implications such as migrant resettlement in the host country.

To date, a predominant focus of research with refugee and asylum seekers has been on inequalities in their health outcomes.^81^ Systematic reviews identify major challenges influencing health care delivery for migrants and refugees and consistently report on the inequalities of health outcomes.^11^, ^12^, ^13^ Challenges in health care delivery that were identified included, but are not limited to, communication, confidence/trust in the provision of care and the continuity of care including resettlement in the host country. Refugees and migrants experience complex challenges in relation to health care, and poorer health outcomes are evidenced by difficulties faced in accessing health care, receiving unequal medical care and appropriate continuity of care.^12^, ^13^ Health outcomes are highly influenced by the quality of health care, and yet, this has received limited attention to date. Quality of health care describes the degree to which it is safe, effective, timely, efficient, equitable and person‐centred.^22^ The first point of patient access to health care is often the primary care of the host country.^12^ Therefore, high‐quality primary care is critical towards redressing inequities. Promoting effective health care delivery for refugees, asylum seekers and their families is a complex challenge. This challenge is faced by both primary care professionals and also patients and families, who face a number of complex hurdles in seeking and accessing care in each phase of the migration and the displacement cycle (i.e., including before and during departure, travel, arrival at destination and possible return).^82^ Moreover, on arrival in a new country of residence, refugees and asylum seekers face several challenges. These include a lack of access or barriers to accessing health care services, patients facing language and cultural differences, high costs, discrimination, administrative hurdles, adverse living conditions and a lack of information about health entitlements, to identify a few.^82^ The majority of the interventions in this review focused on improving the quality of health care within a specific clinical context, although some identified interventions had wider goals to acclimatize and resettle displaced people in the host country.^26^, ^27^, ^34^, ^36^, ^37^, ^40^, ^44^, ^45^, ^46^, ^50^, ^51^, ^57^, ^59^, ^64^, ^67^, ^70^

The vast majority of studies relied on self‐reported data about whether interventions were effective in improving the quality of health care, that is *n* = 51 studies.^25^, ^26^, ^28^, ^29^, ^30^, ^31^, ^32^, ^33^, ^34^, ^35^, ^36^, ^37^, ^38^, ^39^, ^40^, ^41^, ^42^, ^43^, ^44^, ^45^, ^46^, ^47^, ^48^, ^49^, ^50^, ^51^, ^52^, ^53^, ^54^, ^55^, ^56^, ^57^, ^58^, ^59^, ^60^, ^61^, ^62^, ^63^, ^64^, ^65^, ^66^, ^67^, ^68^, ^69^, ^70^, ^71^, ^72^, ^73^, ^74^, ^76^, ^77^, ^79^ Meanwhile, there were fewer large‐scale and longitudinal studies, *n* = 4.^27^, ^57^, ^75^, ^78^ The complexities and challenges of conducting longitudinal studies with humanitarian migrants are identified in the literature.^83^, ^84^ Long‐term and longitudinal research on refugee resettlement is valuable because it can provide an insight into the transformation of challenges and opportunities over time.^84^ All education programmes discussed the importance of being culturally/linguistically relevant in promoting both the physical and psychological well‐being of both receiving and providing care. Studies that explored refugees' experiences were useful in providing an in‐depth understanding of client experiences with the intervention and these were explored in some studies on upskilling refugees/asylum seekers.^54^, ^57^, ^63^, ^66^ The in‐depth understanding of participants' perspectives aided in identifying the subtle, yet critical aspects of care provision that impacted health and well‐being.^54^, ^57^, ^63^ Participants also explained why the positive impact of the intervention waned after the completion of the intervention.^57^ An important aspect was communicating about the implementation of the intervention with the participants.^63^ Research involving resettled refugees and asylum seekers raises methodological and ethical complexities.^84^, ^85^, ^86^ This complex nature of conducting research with humanitarian migrants is reflected in the wider literature and some draw particular attention to methodological issues of sampling, translation and use of local assistants and using an open‐minded approach to draw inferences.^85^ Others have identified ethical considerations in relation to research, its application and policy,^87^ and issues around informed consent, and the notion of do no harm in research.^86^

The broader literature suggests that refugee health requires intersectoral and multidisciplinary work to promote effective health care delivery.^88^, ^89^ The WHO mandates this by advocating for enhanced coordination and collaboration to achieve the goal of universal health coverage for refugees and migrants.^82^ However, this review identified that doctors were predominantly the group provided with the skill development opportunities.^32^, ^37^, ^53^, ^67^, ^69^, ^70^, ^79^ This has implications for involving the wider health care team and interprofessional members in primary care delivery for refugees and asylum seekers. Very few interventions are targeted towards upskilling or encouraging the involvement of interprofessional teams in health care delivery. The potential for enhancing health care quality through interprofessional team involvement requires further exploration. The majority of studies focused on specific issues pertaining to refugee health, with a paucity of interventions that focused on holistic enculturation and adjustment of the displaced people in the new country of residence. Interventions of this nature involved a community approach with the physical–psychosocial–spiritual needs at the centre of focus.^50^, ^59^ There is also an emergence of online resources available for supporting refugee health care that are designed both for primary care doctors and for refugees, but these were often focused on physicians only.^52^, ^67^ The challenge is in managing information available via online resources for refugees and asylum seekers in multiple languages, especially in terms of maintaining its quality and authenticity.^52^ The is an opportunity to explore the role of digital platforms in managing the health of humanitarian migrants and refugees.^90^, ^91^

### 4.1 Implications

The review findings suggest that there is value in involving multidisciplinary health care professionals when exploring models of health care delivery for refugees and asylum seekers, and yet, there is a paucity of interventions involving other members of health care teams beyond doctors. Coordination and integration of health care across different health and nonhealth services have been associated with improved communication and coordination between service providers to meet the needs of migrant patients.^92^ Moreover, the role of the health sector in working across organizations on issues not limited to health but to wider aspects related to migration, social, welfare, education, interior and development sectors is a priority in promoting the health of refugees and migrants.^93^ Refugees are identified to have complex care needs; therefore, a multidisciplinary team is an important mechanism for organizing and coordinating health and care services to meet the needs of individuals with complex care needs.^94^

Papers discussing upskilling of health care professionals highlighted the importance of cultural competence as underpinning quality care for humanitarian migrants.^32^, ^37^, ^53^, ^70^, ^78^, ^79^, ^95^ Time constraints faced by primary health care providers in participating in such activities were identified as a key challenge.^32^, ^37^ Addressing health care professionals' cultural competence is a common approach to improving the quality of health services for culturally and ethnically diverse groups, such as refugees and asylum seekers.^96^, ^97^ A range of individual and organizational approaches to cultural competence in refugee service settings have been identified that comprise different strategies to meet the needs of refugees.^97^ The strategies include individual approaches like developing self‐awareness and cultural competence, along with more organizational strategies like addressing barriers to access and provision of culturally focused care.^97^ Comparisons of the relevance and usefulness of the educational approaches are hampered by the different areas of focus, format and duration of the study. Online approaches are emerging^32^, ^53^, ^67^, ^70^ and can be feasible for health care professionals to access and use, while more targeted approaches need further evaluation in terms of feasibility and efficiency.

#### 4.1.1 Strengths and limitations of the review

A systematic approach to search five different databases with the support of an information scientist led to a comprehensive search strategy and search process. A comprehensive screening approach with a team‐based approach increased the rigour of the selection and extraction processes. The inclusion of only English‐language articles and primary care interventions from the OECD countries means that some relevant material may have been omitted. There was heterogeneity in how the impacts and outcomes were evaluated in different studies. Mostly, studies discussed self‐reported impacts, with a lack of long‐term and longitudinal studies exploring the impacts. Asylum seekers face further uncertainties in relation to seeking refugee status, the possibility of return and unpredictable current legal status.^98^, ^99^ The identified interventions in the primary care setting were designed for both refugees and asylum seekers, and no differentiated intervention specifically for asylum seekers was recognized. A lack of distinction was identified in studies between different types of humanitarian migrants and this identifies a need for more rigorous evaluations, especially those focused on the impact of innovative models on different groups of humanitarian migrants.

## 5 CONCLUSION

This review has identified 55 studies that report on interventions in primary care that were developed to promote effective health care delivery for refugees, asylum seekers and their families. Interventions were designed with a focus on delivering effective, efficient, timely, equitable and person‐centred health care for important issues pertinent to the health of refugees and their resettlement in the host country. Interventions were focused on upskilling humanitarian migrants, their families and health professionals and on models and systems of care to improve health care quality communication and care arrangements. It is identified that there is a paucity of studies that have explored the involvement of a multidisciplinary team and community‐focused and intersectorial approaches that may be important in contributing to quality care provision for this population.

## CONFLICT OF INTERESTS

The authors declare that there are no conflict of interests.

## AUTHOR CONTRIBUTIONS

Maha P. Iqbal, Reema Harrison and Ramesh Walpola wrote the background and developed the methodology. Stephen Mears ran the search and retrieved the final articles. Maha P. Iqbal and Jiadai Li were involved in the screening, data abstraction and analysis. Jiadai Li was involved in data abstraction and quality assessment of the final articles included. Maha P. Iqbal, Reema Harrison, Ben Harris‐Roxas and John Hall wrote the first draft of the article. All authors provided input and have approved the final draft of this article.

## Supporting information

Supporting information.

Supporting information.

## Data Availability

The data that support the findings of this study are available from thecorresponding author upon reasonable request.
