# Supplementary material for: Improving primary health care quality for refugees and asylum seekers: A systematic review of interventional approaches
Source: Health Expect. 2021 Oct 15;25(5):2065–94. doi: 10.1111/hex.13365 (PMC9615090; doi:10.1111/hex.13365)

Database(s): **MEDLINE(R) All including Epub Ahead of Print, In-Process & Other Non-Indexed Citations, Daily and Versions(R)**November 2019 – 01.09.2020

**Search Strategy:**

| **#** | **Searches** | **Results** |
| --- | --- | --- |
| 1 | (refugees or asylum seeker* or undocumented migrant*).mp. | 13553 |
| 2 | exp General Practice/ | 75028 |
| 3 | Primary Health Care/ | 77875 |
| 4 | Community Health Services/ | 31691 |
| 5 | Physicians, Primary Care/ | 3500 |
| 6 | physicians, family/ | 16393 |
| 7 | General Practitioners/ | 7937 |
| 8 | (general pract* or family pract*).mp. [mp=title, abstract, original title, name of substance word, subject heading word, floating sub-heading word, keyword heading word, organism supplementary concept word, protocol supplementary concept word, rare disease supplementary concept word, unique identifier, synonyms] | 135467 |
| 9 | ((family or community or practice*) adj (medic* or doctor$ or physician*)).mp. [mp=title, abstract, original title, name of substance word, subject heading word, floating sub-heading word, keyword heading word, organism supplementary concept word, protocol supplementary concept word, rare disease supplementary concept word, unique identifier, synonyms] | 35392 |
| 10 | (primary adj2 care).tw. | 135683 |
| 11 | or/2-10 | 316512 |
| 12 | 1 and 11 | 826 |
| 13 | limit 12 to english language | 750 |
| 14 | 13 and (201911* or 201912* or 2020*).dt. | 41 |

**NB: .dt. ( The Create Date) in line 14 refers to the date the record was added to PubMed in the yyyymm format. It includes records added in November and December 2019 and all records added in 2020 so far.**


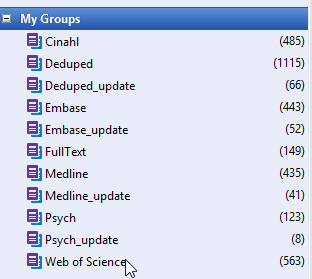

Supplement: Supplementary file 1 — Supporting information. [file HEX-25--s002.docx]
