# Supplementary material for: Improving primary health care quality for refugees and asylum seekers: A systematic review of interventional approaches
Source: Health Expect. 2021 Oct 15;25(5):2065–94. doi: 10.1111/hex.13365 (PMC9615090; doi:10.1111/hex.13365)
Supplement: Supplementary file 2 — Supporting information. [file HEX-25--s001.docx]

| Title | C1-- | C2 | C3 | C4 | C5 | C6 | C7 | C8- | C9 | C10 | C11 | C12 | C13 | Total |
| --- | --- | --- | --- | --- | --- | --- | --- | --- | --- | --- | --- | --- | --- | --- |
| #982 Balachandra_2009 | 1 | 2 | 2 | 2 | 0 | 0 | 0 | 0 | 0 | 1 | 0 | 1 | 1 | 10 |
| #981 Ballard_2018 | 3 | 3 | 3 | 3 | 1 | 2 | 3 | 2 | 2 | 2 | 1 | 2 | 3 | 30 |
| #956 Benjumea-Bedoya-2019 | 1 | 3 | 2 | 2 | 2 | 2 | 2 | 2 | 1 | 1 | 2 | 0 | 1 | 21 |
| #948 Berkson_2014 | 3 | 2 | 2 | 3 | 1 | 3 | 3 | 2 | 1 | 2 | 2 | 1 | 3 | 28 |
| #935 Biegler-2016 | 3 | 3 | 2 | 3 | 3 | 3 | 2 | 3 | 3 | 2 | 2 | 1 | 3 | 33 |
| #931 - Birman 2008 | 2 | 3 | 1 | 2 | 1 | 2 | 1 | 1 | 1 | 1 | 1 | 1 | 3 | 20 |
| #920 - Bonvicini 2019 | 1 | 1 | 2 | 2 | 2 | 2 | 1 | 1 | 1 | 2 | 1 | 0 | 2 | 18 |
| #919 - Borgschulte 2018 | 0 | 3 | 3 | 3 | 2 | 2 | 2 | 2 | 1 | 2 | 2 | 1 | 1 | 24 |
| #918 - Bosson 2017 | 1 | 2 | 2 | 1 | 0 | 1 | 1 | 1 | 1 | 1 | 1 | 1 | 1 | 14 |
| #916 - Bourne 2004 | 1 | 3 | 2 | 1 | 0 | 0 | 0 | 0 | 0 | 0 | 0 | 1 | 1 | 9 |
| #906 - Browne 2018 | 3 | 3 | 2 | 3 | 2 | 3 | 2 | 2 | 1 | 3 | 3 | 1 | 1 | 29 |
| #890 - Bull 2018 | 1 | 3 | 3 | 2 | 1 | 2 | 2 | 1 | 1 | 1 | 1 | 1 | 2 | 21 |
| #877 - Carter 2017 | 0 | 2 | 2 | 3 | 2 | 2 | 2 | 2 | 2 | 1 | 2 | 0 | 1 | 21 |
| #864 - Cheng 2019 | 1 | 2 | 2 | 2 | 1 | 1 | 1 | 2 | 2 | 1 | 1 | 3 | 1 | 20 |
| #855 - Clabots 1992 | 2 | 1 | 1 | 0 | 0 | 1 | 1 | 1 | 1 | 0 | 0 | 1 | 1 | 10 |
| #820 - Culhane-Pera 2005 | 2 | 2 | 2 | 2 | 1 | 1 | 1 | 2 | 2 | 1 | 2 | 1 | 2 | 21 |
| #713 - Duke 2015 | 2 | 3 | 2 | 3 | 1 | 1 | 1 | 1 | 1 | 0 | 1 | 1 | 2 | 19 |
| #702 - Dutcher 2008 | 0 | 1 | 3 | 0 | 0 | 1 | 2 | 0 | 0 | 0 | 1 | 3 | 1 | 12 |
| #1085 - Ekblad 2013 | 3 | 3 | 1 | 2 | 1 | 3 | 2 | 3 | 2 | 1 | 2 | 0 | 2 | 25 |
| #1076 - Esala 2018 | 1 | 3 | 2 | 1 | 1 | 3 | 2 | 1 | 1 | 3 | 2 | 2 | 3 | 25 |
| #1063 - Farokhi 2014 | 0 | 2 | 2 | 2 | 0 | 0 | 0 | 0 | 0 | 0 | 2 | 1 | 1 | 10 |
| #1052 - Ferrari 2016 | 3 | 1 | 1 | 2 | 0 | 3 | 3 | 3 | 2 | 1 | 2 | 0 | 3 | 24 |
| #697 - Gondek 2015 | 2 | 3 | 1 | 3 | 0 | 1 | 3 | 2 | 1 | 1 | 2 | 1 | 2 | 22 |
| #696 - Goodkind 2005 | 3 | 1 | 2 | 2 | 0 | 3 | 3 | 3 | 2 | 3 | 2 | 1 | 3 | 28 |
| #691 - Gould 2010 | 0 | 3 | 1 | 2 | 0 | 1 | 0 | 0 | 1 | 0 | 1 | 0 | 1 | 10 |
| #682 - Grigg-Saito 2010 | 0 | 1 | 1 | 1 | 0 | 0 | 0 | 0 | 0 | 0 | 0 | 1 | 0 | 4 |
| #600 - Jahn 2018 | 2 | 3 | 3 | 2 | 1 | 2 | 1 | 1 | 1 | 1 | 2 | 0 | 2 | 21 |
| #589 - Jirovsky 2018 | 1 | 3 | 1 | 3 | 0 | 3 | 1 | 1 | 2 | 0 | 2 | 0 | 2 | 19 |
| #587 - Johnson 2006 | 2 | 3 | 1 | 2 | 0 | 3 | 1 | 1 | 1 | 1 | 2 | 2 | 1 | 20 |
| #561 - Kennedy 1999 | 2 | 2 | 1 | 3 | 0 | 1 | 1 | 2 | 0 | 2 | 2 | 1 | 2 | 19 |
| #550 - Kirmayer 2003 | 2 | 3 | 1 | 2 | 0 | 2 | 1 | 2 | 1 | 2 | 2 | 2 | 3 | 23 |
| #388 - Martin 2018 | 3 | 3 | 1 | 3 | 0 | 0 | 0 | 0 | 1 | 0 | 0 | 1 | 2 | 14 |
| #378 - McHenry 2016 | 0 | 3 | 2 | 2 | 1 | 2 | 1 | 2 | 2 | 1 | 2 | 0 | 1 | 19 |
| #359 - Michael 2019 | 1 | 2 | 1 | 3 | 2 | 3 | 0 | 3 | 3 | 2 | 3 | 0 | 2 | 25 |
| #1163 - Muller 2020 | 0 | 2 | 1 | 2 | 0 | 2 | 0 | 1 | 1 | 1 | 2 | 3 | 1 | 16 |
| #286 - Njeru 2015 | 2 | 3 | 2 | 3 | 2 | 2 | 3 | 3 | 3 | 2 | 2 | 2 | 2 | 31 |
| #1162 - Northwood 2020 | 3 | 3 | 2 | 3 | 3 | 2 | 3 | 2 | 3 | 2 | 3 | 1 | 3 | 33 |
| #269 - Ong 2010 | 1 | 1 | 2 | 1 | 0 | 0 | 1 | 1 | 1 | 0 | 1 | 0 | 2 | 11 |
| #256 - Parmentier 2004 | 1 | 3 | 1 | 2 | 0 | 0 | 0 | 1 | 1 | 1 | 2 | 1 | 3 | 16 |
| #247 - Percac-Lima 2013 | 1 | 3 | 2 | 3 | 0 | 3 | 2 | 1 | 1 | 1 | 2 | 1 | 2 | 22 |
| #220 - Pottie 2007 | 2 | 3 | 2 | 2 | 0 | 3 | 0 | 0 | 1 | 0 | 2 | 3 | 2 | 20 |
| #210 - Prescott 2018 | 3 | 3 | 2 | 3 | 0 | 3 | 1 | 3 | 1 | 1 | 2 | 2 | 2 | 26 |
| #191 - Reavy 2012 | 3 | 3 | 1 | 3 | 0 | 2 | 1 | 1 | 1 | 0 | 2 | 1 | 1 | 19 |
| #526 - Rodriguez-Torres 2019 | 2 | 3 | 2 | 3 | 0 | 1 | 1 | 2 | 1 | 2 | 2 | 0 | 3 | 22 |
| #490 - Schulz 2014 | 2 | 3 | 1 | 3 | 0 | 2 | 1 | 3 | 1 | 1 | 3 | 0 | 2 | 22 |
| #1148 - Spruijt 2020 | 2 | 2 | 2 | 3 | 0 | 2 | 1 | 3 | 2 | 2 | 2 | 3 | 3 | 27 |
| #142 - Sundquist 2010 | 2 | 3 | 2 | 3 | 1 | 3 | 2 | 3 | 2 | 2 | 3 | 2 | 3 | 31 |
| #122 - Teunissen 2017 | 2 | 3 | 1 | 2 | 2 | 2 | 2 | 1 | 2 | 2 | 3 | 2 | 2 | 26 |
| #1146 - Timlin 2020 | 2 | 2 | 2 | 3 | 3 | 3 | 2 | 2 | 2 | 2 | 3 | 2 | 3 | 31 |
| #67 - Wagner 2015 | 2 | 3 | 2 | 3 | 0 | 2 | 1 | 2 | 2 | 1 | 2 | 1 | 2 | 23 |
| #49 - Weissman 2012 | 2 | 2 | 1 | 2 | 0 | 0 | 0 | 0 | 0 | 0 | 1 | 1 | 1 | 10 |
| #36 - Wieland 2017 | 2 | 3 | 2 | 2 | 0 | 2 | 2 | 2 | 1 | 2 | 2 | 2 | 2 | 24 |
| #29 - Wittick 2018 | 1 | 3 | 1 | 2 | 1 | 2 | 1 | 1 | 1 | 2 | 2 | 0 | 2 | 19 |
| #1139 - Yacoob 2020 | 1 | 3 | 1 | 2 | 2 | 3 | 2 | 3 | 3 | 2 | 3 | 0 | 2 | 27 |
| #10 - Zehetmair 2018 | 2 | 3 | 2 | 3 | 1 | 3 | 2 | 3 | 2 | 2 | 3 | 1 | 3 | 30 |
| Total out of 165 | 90 | 139 | 94 | 125 | 41 | 101 | 74 | 87 | 73 | 67 | 98 | 60 | 106 |  |
|  |  |  |  |  |  |  |  |  |  |  |  |  |  |  |
| Percentage of maximum possible score achieved | 55% | 84% | 57% | 76% | 25% | 61% | 45% | 53% | 44% | 41% | 59% | 36% | 64% |  |
